# Work impairment in bipolar disorder compared to the healthy population: a systematic review

**DOI:** 10.47626/1679-4435-2025-1363

**Published:** 2025-07-13

**Authors:** Régio Marcos Abreu Filho, Caroline Haussman dos Santos, Alberto José Filgueiras Gonçalves

**Affiliations:** 1 Medicina, Faculdade Souza Marques, Rio de Janeiro, RJ, Brazil; 2 Psicologia, Universidade Federal do Rio de Janeiro, Rio de Janeiro, RJ, Brazil; 3 Psicologia, Universidade do Estado do Rio de Janeiro, Rio de Janeiro, RJ, Brazil

**Keywords:** work, bipolar disorder, systematic review, psychiatry, trabalho, transtorno bipolar, revisão sistemática, psiquiatria

## Abstract

Bipolar disorder is a chronic condition that has been insufficiently explored in terms of its predictors of work impairment and its long-term impact on employment. This study aims to compare work impairment in individuals with bipolar disorder to that of a healthy population. A systematic review of the scientific literature was conducted by using PubMed/MEDLINE, SciELO, and PsycINFO databases. Search terms used were: (“bipolar”) and (“work” or “occupational”), with a publication date restriction from 2013 to 2023. A total of 20 articles were selected. All included assessments of employability and work performance using work-specific scales or scales containing work-related items. Most studies showed that a diagnosis of bipolar disorder, along with its long-term effects on behavior, neurocognition, emotional regulation, decision-making, sustained attention, volition, and interpretation of interpersonal events, significantly increases susceptibility to unemployment and dependence on government assistance. Even during periods of clinical stability, maintaining employment at levels comparable to healthy individuals remains highly challenging. Furthermore, the impact of bipolar disorder on employment outcomes appears to be distinct from that of major depressive disorder, schizoaffective disorder, and schizophrenia. Clinical studies consistently indicate that bipolar disorder severely compromises both employability and work performance. However, few effective interventions for improving employability and productivity in this population are currently available.

## Abstract (pt)

O transtorno bipolar é uma doença de longa duração cujos fatores de risco para o comprometimento do trabalho e seus efeitos contínuos no emprego têm sido pouco pesquisados. O objetivo é comparar prejuízo laboral entre pacientes bipolares e a população saudável. Realizou-se uma revisão sistemática de literatura científica sobre o prejuízo do transtorno bipolar no trabalho. Foram utilizadas as bases de dados PubMed/MEDLINE, SciELO e PsycINFO. Os termos de busca empregados foram: (“bipolar”) e (“work” ou “occupational”). Houve restrição quanto ao período de publicação: entre 2013 e 2023. Foram selecionados 20 artigos. Todos avaliaram via escalas laborais ou escalas com itens de avaliação laboral o prejuízo na empregabilidade e desempenho no trabalho dos participantes. Os estudos demonstram em sua maioria que a existência de um diagnóstico de transtorno bipolar e suas consequências de longo prazo em alterações comportamentais, neurocognitivas, habilidades emocionais, de decisão, resumo e foco, volição e diversos campos da interpretação interpessoal e de eventos torna esse paciente muito mais suscetível ao desemprego e auxílio governamental, sendo extremamente difícil mesmo em períodos de estabilidade essa população manter emprego estável em níveis próximos dos controles. Há ainda clara diferença entre impacto e desemprego quando comparado ao transtorno depressivo maior, transtorno esquizoafetivo e esquizofrenia. Os resultados dos estudos clínicos sugerem que o transtorno bipolar provoca grande prejuízo no binômio empregabilidade x desempenho laboral. Em relação ao tratamento e prevenção do prejuízo laboral, os estudos abordam poucas técnicas com eficácia acerca do aumento real da empregabilidade e produtividade.

## INTRODUCTION

Bipolar disorder is a chronic condition that has been insufficiently studied in terms of its predictors of work impairment and its lasting impact on employment.^1^ One of the objectives of this review is to identify key differences in functioning, sociodemographic factors, behavior, and neurocognitive abilities between employed and unemployed individuals with bipolar disorder, as well as in comparison to the general population. Across the selected studies that used neurocognitive and functional assessment scales, there is a consistent and marked distinction in cognitive performance between employed and unemployed individuals, even among those diagnosed with bipolar disorder. Differences are also evident in social functioning and in the way individuals perceive and interact with the world.^2,3^

As demonstrated by several studies in this review, the primary factors contributing to work dysfunction in bipolar disorder are neurocognitive deficits and depressive symptoms. These are strongly associated with poor work attendance, low job satisfaction, and reduced performance.^4^

There is a notable lack of literature and effective interventions specifically targeting work-related disability in individuals with bipolar disorder — particularly among those who remain employed and continue to make long-term contributions.^5,6^ This review aims to address that gap by systematically analyzing original studies from three major databases, offering a broad and updated perspective on the topic.

This systematic review also aimed to expand the understanding of how bipolar disorder affects quality of life and work functioning, particularly during periods between episodes.

## METHODS

We conducted a systematic review on work impairment and employment status in individuals with bipolar disorder, including comparisons with a healthy control. The review was registered in the International Prospective Register of Systematic Reviews, (PROSPERO) under registration number CRD, and followed the Preferred Reporting Items for Systematic Reviews and Meta-Analysis (PRISMA) guidelines for reviews of health-related interventions.

The databases used for the literature search were PubMed/MEDLINE, SciELO, and PsycINFO. The search terms applied were: (“bipolar”) AND (“work” OR “occupational”). Mendeley Reference Manager was used for reference organization. Publications were limited to those published between 2013 and 2023.

The search was conducted independently by two researchers, with discrepancies resolved through consensus. Eligible studies met the following inclusion criteria: (1) original research; (2) inclusion of at least one validated work assessment scale or an evaluation method containing work-related items; (3) a sample size of at least 20 individuals diagnosed solely with bipolar disorder (i.e., without comorbid psychiatric conditions); and (4) clear conclusions regarding work impairment and employability based on the assessment tools used.

Only prospective studies published in English or Portuguese were included. The final search was completed on July 31, 2023.

## RESULTS

### SEARCH AND SELECTION STRATEGY

The initial search yielded 5,042 references in MEDLINE, 647 in SciELO, and 34 in PsycINFO. After removing 633 duplicates across the databases, the remaining references were screened based on their titles and abstracts.

Following the abstract screening, 20 articles were selected from PubMed, three from SciELO, and one from PsycINFO. The main reasons for exclusion at this stage included: the topic not being related to work impairment and bipolar disorder, absence of original findings (i.e., review articles), and lack of work impairment assessment using validated scales.

A total of 24 articles (20 from PubMed, three from SciELO, and one from PsycINFO) were initially selected. Upon a secondary review of abstracts, two articles were excluded due to one or more of the following reasons: sample size of fewer than 20 patients, publication in a language other than English or Portuguese, or use of a retrospective study design.

Thus, 21 articles were shortlisted for full-text review. After reading the full texts, 1 study was excluded because its sample consisted exclusively of individuals with schizoaffective disorder, not bipolar disorder. In the end, 20 articles met all inclusion criteria and were included in the final analysis.

**Figure 1.**
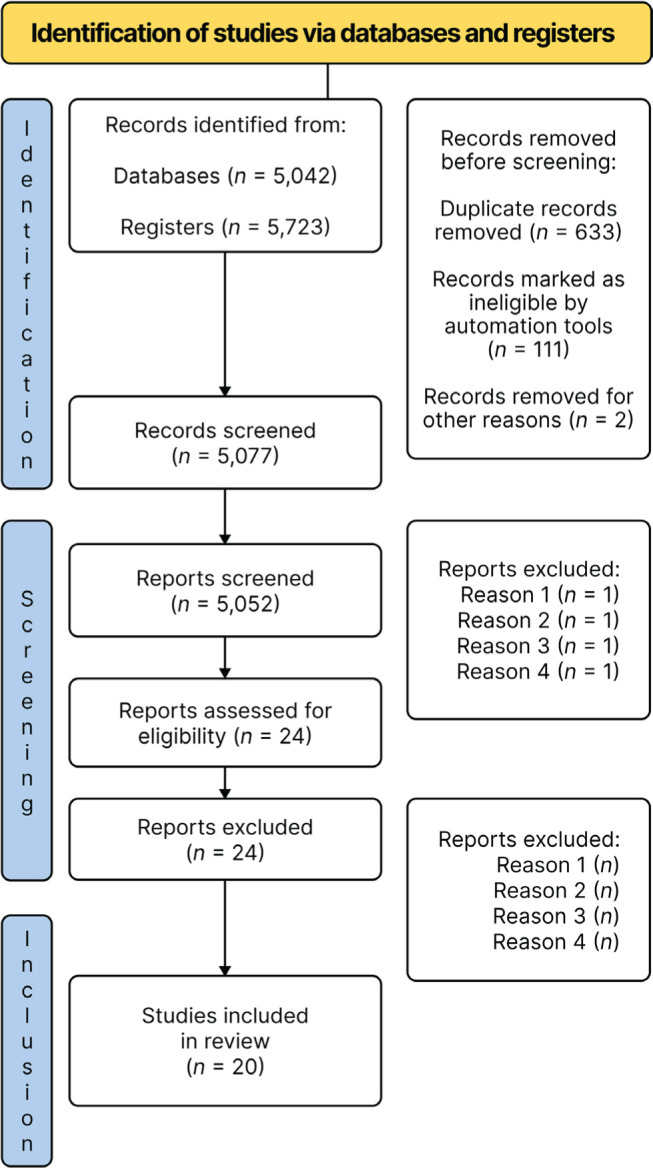
Article search and selection process. Reason 1 = less than 20 patients; Reason 2 = retrospective method; Reason 3 = language other than English or Portuguese; Reason 4 = type of population in sample. Source: Page MJ, McKenzie JE, Bossuyt PM, Boutron I, Hoffmann TC, Mulrow CD, et al. The PRISMA 2020 statement: an updated guideline for reporting systematic reviews. BMJ 2021;372:n71. doi: 10.1136/bmj.n71^7^

Figure 1 presents the flowchart of the article selection process.

Table 1 shows papers about work impairment in bipolar disorder and its results

**Table 1:** Studies on work impairment in bipolar disorder and main findings

Study	Sample	Work assessment instruments	Other instruments	Design	Main results
Arvilommi et al.^8^	n = 90 BD1/101 BD2	SOFAS	SCID-I/P, SCID-II, BDI, YMRS, MDD, PSSS-R	Prediction of disability pension SOFAS and PSSS-R	↑ pension = ↓ SOFAS, ↑ suicidal ideation, ↑ MDD, ↓ PSSS-R
Liu et al.^9^	n = 45 BD1/36 BD2	FAST	HDRS-17, YMRS, TMT-A, MCCB, SDMT, SCWT, BVMT-R, HVLT-R	Comparison between BD1 and BD2 and between employed (E) and unemployed (NE)	BD1 = ↑ FAST; E = ↑ SDMT/HVLT-R; NE = ↓ SDMT, ↓ HVLT-R
Kato et al.^10^	n = 179 BD, 1549 MDD, 27.485 control	WPAI	EQ-5D-5L, PHQ-9, VAS, HRQOL SF-12v2	Comparison between BD, MDD, and controls	TB = ↓ QOL and ↑ PHQ-9; differences between Japan and the USA
Ryan et al.^11^	n = 156 BD/143 control	GAF	SF-36, TSSUS, SSUS, PG/NGT, TMT-A/B, DSWA3, FASCOW, WCST, etc.	Comparison between BD, controls, and E/NE subgroups	BD NE = ↑ episodes, ↓ SF-36, ↑ functional impairment
Schoeyen et al.^12^	n = 226 BD	GAF	CVLT, ISCED, SCID-I, PANSS, IDS-C, YMRS, WASI, NART, PAS	prediction of social benefit	↓ GAF = ↑ benefit; ↑ number of episodes = ↑ benefit
O’Donnell et al.^13^	n = 273 BD	LFQ	DIGS, ASRM	Occupational functioning predictors	↑ PHQ-9 = ↓ LFQ; ↑ cognitive flexibility = ↑ LFQ
Bonnín et al.^14^	n = 107 BD	EEE	WCST, TMT-B, HMRS, YMRS, SCWT, CVLT, FAST, SCID	Functional loss	↑ TMT-B, ↓ WCST = ↑ loss
Echezarraga et al.^2^	n = 120 BD/97 controls	WSAS	QOL	Functional comparison	↑ QOL = ↓ functional loss; ↑ WSAS = ↓ functionality
Yamashita et al.^6^	n = 128 BD	SASS	TMT-B, WCST, SASES, HMRS	Pre and post intervention evaluation	↑ SASS, ↓ TMT-B = ↑ work success
Filia et al.^15^	n = 35 BD (19 E/16 NE)	DIP-DSFM, SOFAS	HAM-D, YMRS, MINI, TEBE	Employment predictors	NE = ↑ internments; E = ↑ SOFAS; identified work barriers and facilitators
Ikenouchi et al.^3^	n = 806 BD	QPE	OR	Stability and employability	↑ episodes = ↑ instability; women = ↑ OR
Karpov et al.^4^	n = 400 (BD, MDD, AD)	SDS	BDI, OASIS, GSE, MSI-BPD	Comparison between diagnoses	↑ BDI, OASIS = ↓ employability
Strassnig et al.^16^	n = 146 SQZ/87 BD	ELE, WHODAS	EFC, EO, WAIS-R	Functional comparison	↑ WHODAS = ↓ employability; better cognition = ↑ insertion
Konno et al.^17^	n = 3137 BD	QE	OR	Employability	↑ episodes = ↑ unemployment; ↑ IQ = ↑ employability
Konstantakopoulos et al.^18^	n = 49 BD/53 control	BPRS, ToM	YMRS, HDRS, WAIS, etc.	Comparison between BD and control	BD = ↓ ToM, ↑ BPRS, HDRS
Vierck & Joyce^5^	n = 36 BD/40 control	SAS	MADRS, CVLT-I, Groton Maze, DSST	Personality assessment	BD = ↑ HA, ↓ SD, ↑ functional dysfunction
Grande et al.^19^	n = 327 BD	FAST	MADRS, YMRS, HARS, QD	Functional predictors	↑ episodes, symptoms axes I/II = ↑ impairment
Tsapekos et al.^20^	n = 80 BD	FAST	TH, FAST, Wechsler, VPA, PDQ, HAMD	Functional dysfunction	↑ FAST, ↑ PDQ, ↑ HAMD = ↑ dysfunction
O’Donnell et al.^21^	n = 237 BD/82 control	LFQ	TN, PHQ-9, SF-12, NEO-PI-R	Employability and stability	BD = ↓ stability; BD with symptoms ↑ = ↓ employability

AD = anxiety disorder; ASRM = Altman Self-Rating Mania Scale; BD = bipolar disorder; BD1 = BD type 1; BD2 = BD type 2; BDI = Beck Depression Inventory; BPRS = Brief Psychiatric Rating Scale; BVMT-R = Brief Visuospatial Memory Test – Revised; CVLT = California Verbal Learning Test; DIGS = Diagnostic Interview for Genetic Studies; DIP-DSFM = demography and social functioning module of the Diagnostic Interview of Psychosis; DSST = Digit Symbol Substitution Task; DSWA3 = Digit Span Working Memory Task; EEE = Epworth Sleepiness); EFC = Executive Function Composite E/NE = employed/not employed; EO = Early Onset (of illness); EQ-5D-5L = EuroQol 5-Dimension 5-Level; FASCOW = Fast Functioning Assessment Short Test; GAF = Global Assessment of Functioning; GSE = General Self-Efficacy Scale; HA = harm avoidance; HAMD = Hamilton Depression Rating Scale 17-item; HAM-D and HDRS = Hamilton Depression Rating Scale; HARS = Hamilton Anxiety Rating Scale; HMRS = Hamilton Mania Rating Scale; HRQOL SF-12v2 = Health-related Quality of Life 12-Item Short-Form Health Survey version 2; HVLT-R = Hopkins Verbal Learning Test – Revised; IDS-C = Inventory of Depressive Symptoms–Clinician Rating; ISCED = International Standard Classification of Education; IQ = intelligence quotient; LFQ = Life Functioning Questionnaire; MADRS = Montgomery and Asberg Depression Rating Scale; MCCB = MATRICS Consensus Cognitive Battery; MDD = major depressive disorder; MINI = Mini International Neuropsychiatric Interview for Bipolar Disorder Studies Version 5.0.0; MSI-BPD = McLean Screening Instrument 123 for borderline personality disorder; NART = National Adult Reading Test; NEO-PI-R = Revised NEO Personality Inventory ; OASIS = Overall Anxiety Severity and Impairment Scale; OR = odds ratio; PAS = Premorbid Adjustment Scale; PANSS = Positive and Negative Symptom Scale; PDQ = Perceived Deficits Questionnaire; PG/NGT = Problem Gambling/Non-Gambling Task; PHQ-9 = Patient Health Questionnaire-9; PSSS-R = Perceived Social Support Scale – Revised; QD = Quality of Daily life (or similar quality of life measure); QE = Quality of Evidence; QOL = quality of life; QPE = Questionnaire for Psychotic Experiences; SASS = Social Adaptation Self-Evaluation Scale; SASES = Spatial Ability Self-Efficacy Scale; SCID = Structured Clinical Interview for DSM; SCWT = Stroop Color and Word Test; SD = self-directedness; SDMT = Symbol Digit Modalities Test; SDS = Sheehan Disability Scale; SF-36 = 36-Item Short Form Health Survey; SOFAS = Social and Occupational Functioning Assessment Scale; SQZ = schizophrenic; SSUS = Social Support at University Scale; TEBE = Test of Everyday Attention for Children; TH = Trait Hope; TMT-A = Trail Making Test Part A; TMT-B = Trail Making Test Part B; TN = Trait Neuroticism (or similar personality trait); ToM = Theory of Mind; TSSUS = Temporal Sensitivity to Social Uncertainty Scale (or similar social cognition measure; VAS = visual analog scale; VPA = Verbal Paired Associates; WAIS-R = Wechsler Adult Intelligence Scale-Revised; WASI = Wechsler Abbreviated Scale of Intelligence; WCST = Wisconsin Card Sorting Test; WHODAS = World Health Organization Disability Assessment Schedule; WPAI = Work Productivity and Activity Impairment questionnaire; YMRS = Young Mania Rating Scale.

### BIPOLAR DISORDER, WORK IMPAIRMENT EVALUATION, AND ASSESSMENT INSTRUMENTS

All 20 selected scientific articles included at least one evaluation scale related to work performance or impairment. This consistency allowed for cross-examination of metrics and repeated evaluation scores across studies, strengthening the statistical robustness and generalizability of our analysis — an essential feature of a systematic review.

Before delving into the specifics of work-related assessment tools and cognitive impairment, it is important to highlight the role of perceived social support, whether at home or in the workplace. This was commonly measured using self-report instruments such as the Perceived Social Support Scale. Findings consistently showed that higher levels of perceived social support were associated with better job performance and greater employment retention.^1,2,13^ This provides a key preliminary insight that will be further explored in the discussion: although individuals with bipolar disorder often experience cognitive impairments and occasional distortions in perception, they are still able to make meaningful inferences about their work ethic, social support, and employability. Notably, their perceived social support closely aligns with actual employment outcomes, as reflected in job retention rates.

Consistent with previous findings, employed individuals with bipolar disorder report higher levels of social support, including support from employers.^6^ In contrast, the same study found that unemployed participants demonstrated lower volition to seek employment and perceived greater barriers to entering the job market. Although these are subjective measures, they are consistent with patterns observed in the unemployed population, thereby reinforcing the hypothesis regarding the role of perceived and structural barriers in work impairment.^6^

The Social and Occupational Functioning Assessment Scale (SOFAS) emerged in this review as a strong predictor of employability. Higher SOFAS scores were associated with lower rates of unemployment and reduced reliance on government pensions.^1,6,13^ This makes SOFAS a robust and coherent parameter for predicting unemployment and assessing social and economic functioning. A recurrent and well-supported finding across studies is that individuals with bipolar disorder exhibit higher unemployment rates and greater dependence on social welfare compared to healthy controls.

Furthermore, it is commonly observed that individuals with bipolar disorder type I (BD-I) spend more time in recovery facilities and experience more frequent manic episodes and crises. This is linked to greater difficulties in social and occupational functioning. Supporting this, patients with BD-I were found to have higher unemployment rates than those with bipolar disorder type II (BD-II), as measured by the frequency of disability pension grants.^1^ Additionally, BD-I patients showed higher scores on the Functioning Assessment Short Test (FAST) compared to patients with BD-II. Since higher FAST scores reflect greater impairment, this further supports the association between BD-I and poorer employment outcomes, as will be demonstrated in subsequent studies reviewed.^2^

As previously discussed, the FAST is a direct correlate of disability pension status. Therefore, it can be reasonably inferred that individuals with bipolar disorder who score higher on the FAST are more likely to be unemployed and to experience broader interpersonal and functional impairments.^2,16,17^ When comparing unemployed and employed individuals with bipolar disorder, the unemployed group typically shows a higher frequency of mood episodes, lower scores on the 36-Item Short Form Health Survey (SF-36), and higher scores on the Global Assessment of Functioning (GAF) scale.^8^ Another independent variable strongly associated with poor occupational outcomes is the number of psychiatric hospitalizations.^13^

A recurring finding in this review is that depressive symptoms play a major role in work impairment among individuals with bipolar disorder. One of the most widely used instruments to assess the impact of depression is the Hamilton Depression Rating Scale (HAM-D). Across several studies, higher HAM-D scores were consistently associated with poorer work performance.^3-6,8,12,13,16,17,20^ In addition to symptom severity, the occurrence of depressive episodes themselves is also negatively correlated with work outcomes.^15,17,19^ The Beck Depression Inventory (BDI), also known as beck-depressive inventory, another validated depression rating scale, further reinforces this association. Studies show that higher BDI scores are statistically linked to increased unemployment in this population.^12^

Although depressive symptoms are the primary contributors, some studies also suggest that manic episodes can negatively affect job retention and acquisition.^15,17,19^ Moreover, frequent mood fluctuations — such as alternating between manic/hypomanic and depressive states — are associated with increased rates of job loss and difficulty maintaining employment.^3^

Other general trends identified across multiple studies suggest that older age is associated with higher rates of unemployment among individuals with bipolar disorder.^1,3-6,8,12,13,17,20^ This may indicate that the longer the natural course of the illness, the greater its cumulative impairments, ultimately leading to social disability. In one study that included a battery of cognitive tests,^3^ healthy controls consistently outperformed individuals with bipolar disorder, reinforcing the hypothesis that the condition is associated with neurochemical and structural brain changes that impair higher-order cognitive functions and information processing abilities. Axis II impairments, which encompass both cognitive deficits and personality disorders, have also been linked to higher rates of unemployment.^17^ The volume of evidence connecting cognitive dysfunction in bipolar disorder to employment difficulties is substantial and increasingly clear.

A novel finding from one study^19^ is that mixed episodes were not significantly correlated with unemployment. This could be due to statistical error, sampling bias, or randomness; however, it also raises the possibility that mixed features — combining depressive and manic/hypomanic symptoms — may partially offset certain functional impairments, potentially preserving interpersonal performance and the ability to maintain employment.

In one of the studies, a score of 70 on Part B of the Trail Making Test (TMT-B) was established as the cutoff point for successful participation in a rehabilitation program.^5^ Thus, lower TMT-B scores were associated with greater occupational success among participants. The TMT-B is one of several instruments used to assess cognitive functioning, and its findings reinforce the consistent evidence of cognitive impairment in individuals with bipolar disorder. Supporting this, additional studies have shown that unemployed patients tend to have significantly lower scores in the autonomy domain.^2^

### BIPOLAR DISORDER X SCHIZOPHRENIA X DEPRESSIVE DISORDER JOB STATISTICS/DISABILITY PENSION

Individuals with bipolar disorder generally have higher employment rates than those with schizophrenia.^9^ In the same study, a comparison between psychotic bipolar patients and healthy controls revealed that controls were significantly more likely to be employed.^11^ The World Health Organization Disability Assessment Schedule (WHODAS) was used to evaluate both bipolar and schizophrenic patients, with the aim of correlating housing status (homeless or not) and employability. Among individuals with bipolar disorder, no clear relationship was found between WHODAS scores and housing status. However, among those with schizophrenia, a strong correlation was observed — higher WHODAS scores were associated with homelessness and lower employability.^11^

According to one study, only 5.3% of patients with schizophrenia, 29.3% of those with bipolar disorder, and 33% of individuals with depressive disorder are employed full-time.^12^ Additionally, unemployed individuals with bipolar disorder scored higher on the Sheehan Disability Scale (SDS). Compared to individuals with depressive disorder, those with bipolar disorder also tend to have more frequent hospital visits.^13^ This likely reflects the more severe course of bipolar disorder, characterized by more intense episodes, greater cognitive impairment, increased suicide risk, and longer durations of illness episodes.

## DISCUSSION

### OVERVIEW

This systematic review examined scientific studies investigating the negative impact of bipolar disorder on work performance and employability. Only studies with samples of at least 20 individuals who were exclusively diagnosed with bipolar disorder were included. All of the studies used validated assessment scales to measure functional impairment.

While widely used tools such as the SOFAS and the FAST provide valuable insights, they also present limitations that warrant further investigation. For instance, do these instruments account for cultural differences in how work performance is expressed and evaluated? Are they equipped to capture the nuances of nontraditional work arrangements?

Additionally, although several scales incorporate elements related to social support and interpersonal networks, the measurement of social support remains largely subjective. This raises important questions: how can we objectively evaluate the impact of social support on work functioning in bipolar patients, especially when the disorder itself may distort perception? A severely ill patient with a strong support system might perceive it as insufficient, while a high-functioning patient with limited support might still be able to maintain stable employment. How can we quantify these differences, and is it even possible to establish a reliable correlation between perceived and actual social support in relation to occupational outcomes?

Developing alternative frameworks that assess not only cognitive deficits but also residual cognitive strengths and compensatory strategies used by individuals with bipolar disorder is essential. In addition, this review underscores the significant negative impact of depressive episodes on work functioning. Future research should aim to distinguish the specific effects of different mood states — mania, hypomania, mixed episodes, and depression — on occupational performance, while considering the possibility of non-linear or interactive relationships. Notably, only two studies included in this review attempted to directly compare the impact of these distinct mood states on work performance.

This review highlights the critical role of social support in promoting work retention among individuals with bipolar disorder. However, a broader discussion requires a deeper understanding of which specific types of support are most beneficial. Is instrumental support (e.g., job coaching) more effective than emotional support (e.g., peer groups or empathetic colleagues)? Can work environments be intentionally structured to foster supportive conditions that complement individual support systems? Furthermore, examining the potential buffering effect of social support on the relationship between mood episodes and occupational functioning could yield valuable insights.

This review suggests a difference in employment rates between individuals with bipolar disorder type I and type II. To inform more effective clinical management, a more granular analysis is needed. Are there distinct cognitive or symptomatic profiles associated with each subtype that differentially affect occupational functioning? For instance, could the rapid cycling often observed in bipolar disorder type II contribute to unique challenges in maintaining employment? Investigating these questions may support the development of more targeted interventions, allowing treatment strategies to be better tailored to the specific functional needs of each bipolar subtype.

The current review primarily focuses on cross-sectional data. Longitudinal studies that track the employment trajectories of individuals with bipolar disorder are essential to gain a more comprehensive understanding of how the illness impacts work across different phases, including periods of recovery and relapse. Moreover, the lack of intervention data highlights a critical gap that requires urgent attention.

A key area for future research involves the development and testing of targeted interventions that address both the cognitive and social dimensions of work impairment. Such interventions could include cognitive rehabilitation programs designed to enhance work-related cognitive abilities as well as mindfulness-based approaches to help manage stress and improve emotional regulation — both of which are essential for sustained occupational functioning.

Speaking of digging deeper, cognitive deficits definitely play a role — but I want to know which specific areas are most affected. Is it executive function that interferes with organization? Or is it working memory, making it difficult to manage multiple tasks? And how do these issues translate into real-world challenges at work? Once we understand that, we can start thinking about possible interventions. If we could target those specific cognitive areas with training programs, maybe we could help people with bipolar disorder thrive in the workplace.

These questions remain unanswered in this review — and will likely stay that way for years to come. Research on these topics is still limited, and we probably need a deeper understanding of the brain as a whole before we can properly address these issues. For now, we are mostly limited to general interventions with non-specific targets.

Furthermore, exploring the neurobiological underpinnings is essential. Brain imaging and neurochemical studies could help clarify the neural mechanisms that link bipolar disorder to work-related dysfunction. This knowledge could guide the development of targeted interventions aimed at specific neural pathways. Overall, this review provides a solid foundation for understanding the connection between bipolar disorder and work impairment.

Developing targeted interventions tailored to the specific needs of individuals with bipolar disorder — across its various subtypes — is crucial for improving both work outcomes and overall quality of life. Advancing this research agenda holds the potential not only to reduce work-related impairment but also to promote social inclusion and economic empowerment for those living with bipolar disorder.

## CONCLUSIONS

The clinical studies reviewed here indicate that bipolar disorder significantly impacts employability and the ability to maintain long-term employment when compared to healthy controls. The primary contributing factors are not limited to the acute phases of mania and depression, as previously assumed. Instead, the studies highlight a combination of social difficulties, neuropsychological impairments, cognitive deficits across multiple domains, heightened social stress, and altered emotional intelligence. These factors contribute to reduced workplace functioning and difficulty sustaining job performance, ultimately leading to job loss or inability to maintain stable employment.

This pattern of cognitive impairment makes unemployment and underemployment a long-term issue for individuals with bipolar disorder — even during periods of mood stability between episodes. It is a particularly difficult problem to address, especially for patients without family support or strong social networks to provide financial help. Many public health services are unavailable or insufficient, and patients often need to cover the cost of medications, therapy, hospital stays, and other expensive treatments out of pocket. As a result, improving work conditions, increasing job stability, and securing higher wages could help reduce these financial burdens by minimizing periods of underemployment and unemployment, ultimately providing more disposable income to support ongoing treatment needs.

A greater level of evidence is also needed regarding interventions implemented during the course of the disorder, particularly in its early stages. Early effective intervention may help mitigate the long-term socioeconomic impact of bipolar disorder. However, to date, evidence supporting such targeted approaches remains limited and scarce in literature.
